# Excessive Trabeculation of the Left Ventricle: *JACC: Cardiovascular Imaging* Expert Panel Paper

**DOI:** 10.1016/j.jcmg.2022.12.026

**Published:** 2023-03

**Authors:** Steffen E. Petersen, Bjarke Jensen, Nay Aung, Matthias G. Friedrich, Colin J. McMahon, Saidi A. Mohiddin, Ricardo H. Pignatelli, Fabrizio Ricci, Robert H. Anderson, David A. Bluemke

**Affiliations:** a William Harvey Research Institute, National Institute for Health and Care Research Barts Biomedical Research Centre, Queen Mary University London, London, United Kingdom; b Barts Heart Centre, St Bartholomew’s Hospital, Barts Health National Health Service Trust, London, United Kingdom; c Department of Medical Biology, Amsterdam Cardiovascular Sciences, University of Amsterdam, Amsterdam University Medical Center, Amsterdam, the Netherlands; d Department of Medicine, McGill University Health Centre, Montreal, Quebec, Canada; e Department of Diagnostic Radiology, McGill University Health Centre, Montreal, Quebec, Canada; f Department of Paediatric Cardiology, Children’s Health Ireland at Crumlin, Dublin, Ireland; g Department of Pediatric Cardiology, Texas Children’s Hospital, Houston, Texas, USA; h Department of Neuroscience, Imaging, and Clinical Sciences, “G.d’Annunzio” University of Chieti-Pescara, Chieti, Italy; i Biosciences Institute, Newcastle University, Newcastle, United Kingdom; j School of Medicine and Public Health, University of Wisconsin, Madison, Wisconsin, USA

**Keywords:** cardiovascular imaging, clinical management, diagnosis, etiology, left ventricular noncompaction, prognosis, CMR, cardiac magnetic resonance

## Abstract

Excessive trabeculation, often referred to as “noncompacted” myocardium, has been described at all ages, from the fetus to the adult. Current evidence for myocardial development, however, does not support the formation of compact myocardium from noncompacted myocardium, nor the arrest of this process to result in so-called noncompaction. Excessive trabeculation is frequently observed by imaging studies in healthy individuals, as well as in association with pregnancy, athletic activity, and with cardiac diseases of inherited, acquired, developmental, or congenital origins. Adults with incidentally noted excessive trabeculation frequently require no further follow-up based on trabecular pattern alone. Patients with cardiomyopathy and excessive trabeculation are managed by cardiovascular symptoms rather than the trabecular pattern. To date, the prognostic role of excessive trabeculation in adults has not been shown to be independent of other myocardial disease. In neonates and children with excessive trabeculation and normal or abnormal function, clinical caution seems warranted because of the reported association with genetic and neuromuscular disorders. This report summarizes the evidence concerning the etiology, pathophysiology, and clinical relevance of excessive trabeculation. Gaps in current knowledge of the clinical relevance of excessive trabeculation are indicated, with priorities suggested for future research and improved diagnosis in adults and children.

## Highlights

• Current evidence shows that the extent of trabeculated vs compact myocardium in the adult is determined by differential (allometric) growth of each myocardial layer. Because trabeculated myocardium does not coalesce to form the compact myocardial wall, the term “left ventricular noncompaction” is inaccurate and use should be discouraged.
• Excessive trabeculation, as diagnosed using existing criteria, may be present as a normal variant or as a response to preload conditions.
• In adults incidentally found to have excessive trabeculation with normal myocardial function and morphology, clinical management is determined by other cardiovascular symptoms or abnormalities, without regard to trabecular pattern.
• In adults diagnosed with hypertrophic or dilated cardiomyopathy where excessive trabeculation is also present, the extent of ventricular trabeculation has not been demonstrated to alter management nor prognosis. Following guidelines developed specifically for those conditions is recommended.
• In infants and children, caution is warranted because fewer data are available regarding the etiology and prognosis of excessive trabeculation. Clinical evaluation for occult neuromuscular disease or another genetic/metabolic etiology may be appropriate.

Excessive trabeculation is a ventricular phenotype identified by imaging studies, most frequently echocardiography and cardiac magnetic resonance (CMR).^1^ Similar to ventricular wall thickness or diameter, the presence of excessive trabeculation by itself does not define the presence of cardiomyopathy. As pointed out by Jenni, Oechslin, and colleagues,^2^^,^^3^ excessive trabeculation may be a normal variant or a physiological response to conditions of increased preload or afterload, such as pregnancy or athletic participation.^4^^,^^5^ Because of the considerable variation in extent of ventricular trabeculation in the population, individuals with excessive trabeculation in isolation may pose diagnostic and management challenges.^6^ For example, excessive trabeculation is reported to be associated with some pathological conditions, including well-recognized heart muscle disorders. In such cases, it frequently remains unclear whether the phenotype itself identifies a very specific (and rare) cardiomyopathy (ie, so-called left ventricular noncompaction cardiomyopathy) or represents a secondary response to altered preload and/or afterload in patients with myocardial dysfunction.^7^^,^^8^ We list the references to so-called noncompaction in the current international guidelines in the Supplemental Table 1.

In this expert consensus paper, we summarize the published reports relevant to excessive trabeculation and its association with cardiomyopathy in both children and adults (see Supplemental Figure 1 for publication scale and trends). Except for historical context, we avoid the term “noncompaction” because new developmental biology research shows that the term misrepresents the nature of fetal development of the ventricular walls. We use the term “excessive trabeculation” when left ventricular trabecular morphology exceeds previously described thresholds. In addition, we offer considerations for the management of patients in a variety of situations with excessive trabeculation.

## Early Published Reports of Left Ventricular Noncompaction Cardiomyopathy

The early medical published reports on excessive trabeculation may be divided into 2 areas of research. The first involved infants or young children with marked ventricular trabeculation in association with congenital heart disease, which was often fatal. The second concerned adults with cardiomyopathy of unknown origin and/or arrhythmias, again with marked trabeculation of the left ventricle. In infants, the deep endomyocardial spaces found with exaggerated trabeculations, often called sinusoids, were suggested to be secondary to excessive intracavitary pressures during embryonic and fetal development in association with structural heart disease.^9^ The finding in adults, in contrast, was proposed to be secondary to an arrest of a presumed process of compaction of embryonic trabeculations to form the compact ventricular walls.^10^ As we will emphasize, recent data on embryogenesis do not support this concept. Nonetheless, large series using echocardiography in children and adults have estimated prevalence of so-called noncompaction cardiomyopathy between 0.02% and 0.14%.^11^, ^12^, ^13^, ^14^ The estimates, however, are limited by selection bias, varying definitions of excessive trabeculation, and an unclear relationship to contemporary views regarding myocardial development.

## Definitions of Excessive Trabeculation

Several quantitative definitions for excessive trabeculation have been proposed (Table 1). Most frequently, echocardiographers use the criteria published by Jenni et al.^3^ These include, first, the presence of a 2-layered myocardium; second, a ratio of trabecular to compact myocardium >2:1 measured in end-systole in the short-axis view. The typical location of the most pronounced trabeculation in the mid lateral, apical and mid inferior segments and the absence of coexisting cardiac abnormalities is presumed. In a recent meta-analysis seeking to assess the prevalence of so-called noncompaction,^15^ almost two-thirds of published echocardiographic studies used criteria that included trabecular to compact myocardium >2:1 at end-systole. Using this reference, prevalence among 23 cohorts was 0.56%.^15^ To our knowledge, prevalence of excessive trabeculation, at 0.076%, has been assessed in only 1 population-based neonatal echocardiographic study.^16^

**Table 1:** Examples of Echocardiographic and CMR Approaches to Determining the Extent of LV Trabeculations

	Jenni et al^3^	Petersen et al^17^	Jacquier et al^105^	Stacey et al^139^	Captur et al^140^
Modality	Echocardiography	CMR	CMR	CMR	CMR
Sample size	Noncompaction (n = 34) No control group	Noncompaction (n = 7) Control subjects (n = 170)	Noncompaction (n = 16) Control subjects (n = 48)	Noncompaction (n = 122) No control group	Noncompaction (n = 30) Control subjects (n = 105)
Study design/external validation	Retrospective/no external validation cohort	Retrospective/no external validation cohort	Retrospective/no external validation cohort	Retrospective/no external validation cohort	Retrospective/no external validation cohort
Definition of noncompaction	Absence of coexisting cardiac disease Numerous excessively prominent trabeculations and deep intertrabecular recesses Intertrabecular spaces filled by direct blood flow from the ventricular cavity, on color Doppler imaging	Bilayered appearance on echocardiography combined with increased pretest probability (eg, similar appearance in first-degree relatives, associated neuromuscular disorder, or complications, such as systemic embolization and regional wall motion abnormalities)	Diagnosis of noncompaction was established on echocardiographic criteria	Consecutive patients from CMR reports that mention trabeculation or noncompaction	Diagnosis of noncompaction on echocardiographic criteria and at least 1 of the following: positive family history, associated neuromuscular disorder, regional wall motion abnormality, noncompaction-related complications (arrhythmia, heart failure, or thromboembolism)
Description	Noncompaction to compaction ratio Decreased thickening and hypokinesia present within, but not limited to, the noncompacted segments	Two-layered myocardium Measured at the most pronounced trabeculations, avoiding apex Measurement perpendicular to compact myocardium	Short-axis cines for total LV mass and compact mass to define trabecular mass Papillary muscle included in the myocardial mass	Apical short-axis views 16-24 mm from the true apical slice Region with the largest noncompaction to compaction ratio	Loss of base-to-apex fractional dimension gradient
Cardiac phase	End-systole	End-diastole	End-diastole	End-systole	End-diastole
Cardiac view	Short axis	Long axes (4-chamber, 2-chamber, 3-chamber)	Short-axis stack	Apical short axis	Short-axis stack
Excessive trabeculation cutoff	Noncompaction to compaction ratio >2	Noncompaction to compaction ratio >2.3	Trabecular mass >20%	Noncompaction to compaction ratio ≥2	Fractal dimension ≥1.30

These definitions highlight variation in current definitions of excessive trabeculation. Because imaging studies are typically needed to define disease presence without other independent standard of reference, inclusion bias is typically present in such studies. Note that “noncompaction” refers to terms in the original references, rather than the more contemporary description of excessive trabeculation.

CMR = cardiac magnetic resonance; LV = left ventricular.

CMR has increasingly been used to characterize myocardial disorders. Compared to echocardiography, CMR has greater contrast resolution and blood-muscle differentiation, allowing better visualization of ventricular trabeculation. Like echocardiography, several criteria have been suggested (Table 1), with the one proposed by Petersen et al^17^ most frequently applied. This criterion requires a ratio of the trabecular to compact myocardial thicknesses >2.3 at end-diastole in long-axis views. Cardiac computed tomography and, to a lesser extent, invasive ventriculography can also visualize left ventricular trabeculation. However, there are no generally accepted morphologic diagnostic criteria for either technique.

Excessive trabeculation measured by current criteria occurs in individuals without cardiomyopathy. When applied to general populations, the Petersen criteria were met in about 20% of participants in 5 population-representative cohorts.^15^ In the MESA (Multi-Ethnic Study of Atherosclerosis), 43% of participants who were asymptomatic without cardiac disease or hypertension met the Petersen criteria in at least 1 myocardial segment.^1^ Investigators have, therefore, also considered parameters such as the relative or absolute thickness and mass of the trabecular and compact layers,^18^ the number of segments affected,^19^ the location of the affected segments,^20^ the trabecular morphology,^21^ and the morphology of the papillary muscles.^22^

It is also possible that conventional descriptors are too simplistic to capture our visual impression of excessive trabeculation. The CMR fractal dimension is an example of a sophisticated and highly reproducible mathematical scoring of endocardial complexity ranging from 1 (a straight line) to 2 (complete filling of the 2-dimensional space contained by the ventricular trabeculation).^23^ In MESA,^24^ fractal dimensions were larger in individuals with hypertension, greater left ventricular wall thickness, and greater left ventricular mass. African American participants had greater fractal dimensions than White participants did. In this regard, the fractal dimension provided insight into factors now understood to result in greater trabecular thickness. However, in 700 patients referred for CMR, high fractal dimension was present in 23 patients and was also indeterminate in predicting cardiovascular events.^25^ Specific functional indices proposed to diagnose or risk stratify patients with latent or overt cardiomyopathy with excessive trabeculation are attractive because they target the underlying functional disturbance.^26^, ^27^, ^28^, ^29^ They do not, however, provide evidence of causality to a possible morphologic appearance of excessive trabeculation.

## The Embryology of Ventricular Development; Noncompaction as a Misnomer

**Figure 1:**
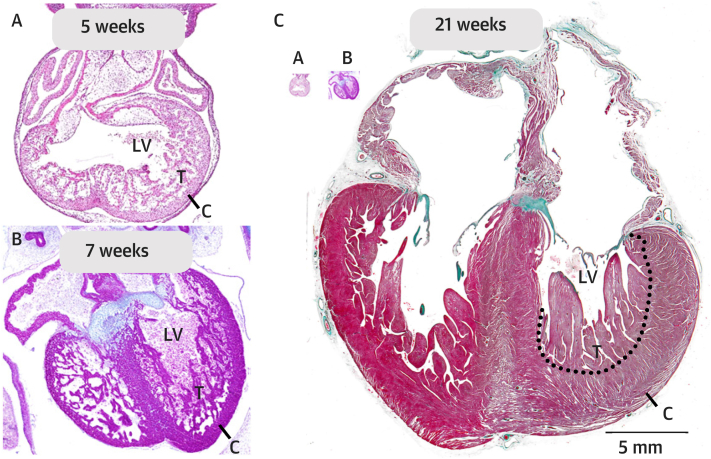
Developmental Anatomy of the Human LV **(A)** At 5 weeks gestation, Carnegie stage 14 is shown; a substantial trabeculated **(T)** wall has developed; and the compact wall **(C)** is thin. **(B)** At 7 weeks gestation, Carnegie stage 19 is shown, and the ventricular wall remains much trabeculated. **(C)** At 21 weeks gestation, the heart (and fetus) has grown tremendously, notice the images of the 2 embryonic hearts of **A** and **B** are inserted to scale. The compact wall is now much thicker than in the embryonic stages and so is the trabecular layer. This illustrates that a decrease of the trabecular layer, that is, compaction, is not required for the formation of a thick compact wall. LV = left ventricle.

**Figure 2:**
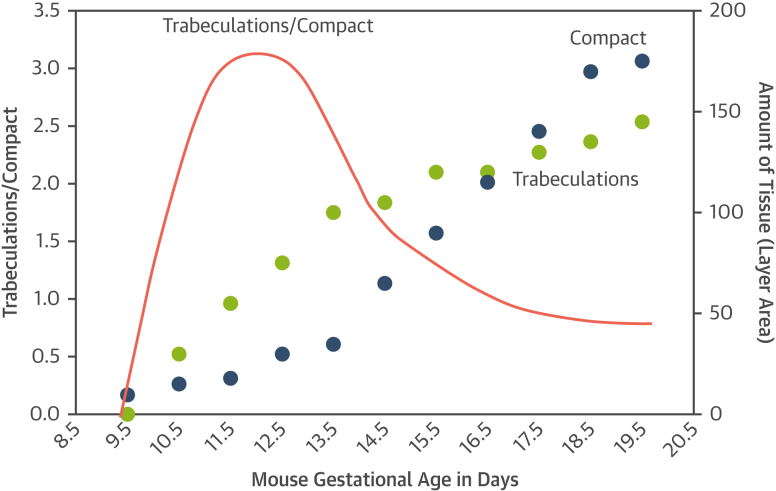
How Development Shapes LV Wall Morphology Developmental changes to the proportions of trabecular and compact myocardium **(red line)** are driven by different rates of positive growth, not compaction, of the trabeculations **(green dots)** and compact tissue **(blue dots)** that vary by gestational age. Such differing rates of growth of different parts of the body is termed “allometric growth” and is a frequent driver of morphologic change in development and in nature. The graphs are redrawn based on data from a mouse model.^141^ Abbreviation as in Figure 1.

In normal development, the trabecular parts of the ventricles are known to balloon out from the outer curvature of an initial primary tube,^30^^,^^31^ with the trabeculations forming secondary to signaling from the endocardium.^32^, ^33^, ^34^ By the fifth week of gestation, the ventricular walls are extensively trabeculated (Figure 1A). Over the subsequent embryonic, fetal, and postnatal periods of development, the heart grows many-fold (Figure 1).^35^^,^^36^ During these periods, both the trabecular and the compact myocardial layers also grow many-fold, but not always in equal proportion (Figure 1).^37^^,^^38^ The ratio of thicknesses between the layers decreases during development, despite an increase in the volume of both, indicating greater growth in the compact than the trabecular layer (Figure 2). These morphometric observations are amply supported by pulse labeling and immunohistochemical studies, which show greater proliferation of cardiomyocytes in the compact wall than in the layers making up the trabeculations.^39^, ^40^, ^41^, ^42^ When proliferation is inhibited experimentally in the trabecular layer, compact mural thickness is largely unaffected.^43^ The compact wall is also able to form normally even when excessive trabeculation is induced by suppression of NKX2-5.^44^ For its normal development, therefore, growth of the compact wall is largely independent of that in the trabecular layer.

Differing rates of growth of different parts of the body is termed “allometric growth.” Allometric growth is a prevalent driver of morphologic change and development in nature.^45^ In this regard, contemporary observations that indicate a continuous positive growth of the trabecular and compact myocardial layers are in direct opposition to earlier notions suggesting that the compact layer is formed as a result of “compaction” of pre-existing trabeculations.^9^^,^^46^ The presumed process of “compaction” has been considered to represent an “intrauterine arrest.”^10^^,^^47^^,^^48^ There is currently no evidence of which we are aware to support this notion. It follows that the term “noncompaction” has no foundation in myocardial development.^38^^,^^49^^,^^50^

## Molecular Biology of the Developing Myocardium

Studies of developing human and animal hearts have shown that most cardiomyocytes of the trabecular layer initially have a different molecular identity from those in the neighboring compact layer.^31^ Later, during the fetal and postnatal periods, cardiomyocytes in trabecular and compact layers have mostly achieved similar identities;^51^ the human trabecular layer becomes vascularized. Even in humans with excessive trabeculation, trabecular cardiomyocytes have lost their embryonic identity and are much more akin to those of the compact wall.^52^^,^^53^ Experimental models of excessive trabeculation are discussed in the Supplemental Appendix.

Histological investigations of individuals with cardiomyopathy and with excessive trabeculation show intramyocardial fibrosis to be a common finding.^54^ The pattern of fibrosis, however, varies substantially from case to case.^22^^,^^55^ In this regard, direct comparisons made between the cardiomyocytes of the trabecular layer and their neighboring compact cardiomyocytes are largely missing.^54^ Greater hypertrophy of the cardiomyocytes in the trabecular than compact layers, nonetheless, has been reported in explanted hearts.^56^ Therefore, evidence to date suggests that trabecular and compact cardiomyocytes are similar, although differences may exist in their responses to different pathologic and physiological processes.

## Determinants and Associations of Excessive Trabeculation

### Genetic determinants

In the community-based UK Biobank CMR study, left ventricular trabeculation measured in fractal dimension was observed to have ∼20% heritability.^57^ That genome-wide study identified 16 genome-wide loci harboring genes regulating cytoskeletal arborization that associated with trabecular complexity. Several loci contained Mendelian genes associated with cardiomyopathy, such as *TTN*, *TNNT2*, and *PLN*. Loci associated with lower fractal dimension conferred higher risk of dilated cardiomyopathy and heart failure in both observational and Mendelian randomization analyses. This suggests a potential role of normal trabecular structure in maintaining cardiac output, or alternatively, that observed associations with certain genetic forms of cardiomyopathy and excessive trabeculation may be mediated by the roles that these genes have in development.

For patients in whom so-called noncompaction is diagnosed, causative genetic sequence variations are reported in approximately one-third of individuals, although these estimates are confounded by inclusion bias. Such biases include patient age, family screening, heterogeneity in case ascertainment, variability in content and size of genetic screening panels, and inconsistent interpretation of the pathogenicity of genetic variants.^58^ When a genetic cause was suspected, autosomal dominant transmission was most frequently reported, although X-linked recessive, autosomal recessive, and mitochondrial inheritance have also been described.^59^

Gene sequence variation associated with so-called noncompaction were recently evaluated in several systematic reviews.^58^^,^^60^^,^^61^ In determining the relevance of genetic associations, these studies also demonstrated the critical importance of case ascertainment. For example, left ventricular dilation and/or systolic dysfunction were present in more than three-fifths of the cases studied. Of the included individuals, one-tenth had congenital heart disease. Left ventricular hypertrophy was also frequent.^58^^,^^60^ The mutant genes identified in these patients had also been associated with well-known cardiomyopathies and congenital cardiac malformations.

In 2 of these recent systematic reviews,^58^^,^^60^ a wide range of genes encoding sarcomeric, cytoskeletal, mitochondrial, desmosomal storage, and ion channels, proteins were linked with a phenotype that includes excessive trabeculation.^62^ Notably, in one of these studies, abnormalities in *MYH7*, *MYBPC3*, *ACTC1*, and *TTN* represented just over two-fifths of the identified sequence variations.^58^ Similar themes were identified in the other large systematic review.^60^ Case ascertainment was variable and incomplete, and the associated genes again suggested that cases could represent phenotypic variants of hypertrophic or dilated cardiomyopathies. Truncating variants in *MYH7*, *ACTN2*, and *PRDM16* were detected in some cases, but such truncating variants have not been identified as causes of more specific cardiomyopathies, suggesting that they may associate more specifically with excessive trabeculation.^60^ *PRDM16* has been shown to be crucial for normal mural development in mouse models.^63^

Due to the aforementioned considerations, contemporary guidelines advocate genetic testing according to the presence of the features of a conventional cardiomyopathy,^64^, ^65^, ^66^, ^67^ rather than when the phenotypic feature of excessive trabeculation is incidentally detected in patients who are asymptomatic with otherwise normal cardiac findings.^68^

### Associations with neuromuscular disease

Excessive trabeculation has been observed in several neuromuscular disorders, including specific genetically determined conditions such as Barth syndrome,^69^ mitochondrial disorders,^70^ nuclear envelopathies,^71^ dystrobrevinopathy,^72^ myotonic dystrophy, zaspopathy,^73^ and myoadenylate deaminase deficiency,^74^ as well as Duchenne and Becker types of muscular dystrophy.^75^ A causal relationship with the underlying genetic defects, however, has yet to be established, with genotypic-phenotypic heterogeneity largely unexplained.^54^^,^^76^ The combination of phenotype and neuromuscular disease, nonetheless, may have clinical and prognostic implications.^77^ In a large, single-center, prospective study in which excessive trabeculation was defined using the Stöllberger criteria, almost 80% of the patients who were neurologically examined were found to suffer from a neuromuscular disorder.^78^ Neuromuscular disorders of specific or unknown etiology, furthermore, were an independent predictor of all-cause death.^54^^,^^78^ Excess mortality was attributed to rhythm and conduction system disorders, respiratory muscular involvement, higher prevalence of cardiovascular autonomic dysfunction, and reduced mobility.^54^^,^^79^ A high prevalence (20%) of cardiomyopathy with excessive trabeculation was reported prospectively in a large cohort of patients with Duchenne and Becker types of muscular dystrophy assessed using echocardiographic criteria.^75^

A retrospective analysis of CMR scans of another large cohort with Duchenne muscular dystrophy showed almost 30% of patients had excessive trabeculation in at least 1 cardiac segment when using Petersen criteria.^1^^,^^80^ Longitudinal analysis of serial studies on a subgroup of patients with Duchenne muscular dystrophy documented a rate of change in the trabecular to compact ratio of +0.4 per year.^80^ This reflected both a progressive increase in the thickness of the trabecular layer, as well as progressive thinning of the compact wall. The investigators suggested these observations supported a concept of dystrophin cardiomyopathy as a progressive disease characterized by a fragile cytoskeleton, leading to worsening left ventricular systolic function and compensatory remodeling of the trabecular myocardium over time.^80^^,^^81^

### Pregnancy

Reversible excessive trabeculation is known to develop secondarily to increased preload in a sizeable proportion of individuals who are pregnant with otherwise normal hearts. The feature had usually resolved by 12 weeks subsequent to delivery, although with some variability in the regression of the trabecular layer, this being similar to the variation in the reduction of the overall left ventricular mass.^5^ African American women were 3 times more likely to develop such features during pregnancy than were Caucasian women.^5^ This is suggestive of a possible underlying genetic susceptibility in the adaptive response of the myocardium to volume and pressure overload.^82^

### Exercise

A reversible phenotype of excessive trabeculation has been reported in athletes. This is recognized as a morphologic epiphenomenon related to high cardiac preload demand associated with intensive physical exercise.^83^ The prevalence of ratios fulfilling the excessive trabeculation criteria among competitive athletes by echocardiography ranges between 1.4% and 8.1%. This varies according to different definitions, ethnicities, and the specific sports disciplines.^4^^,^^84^

In a younger (mean age 48 years), community-based cohort of physically active participants involved in the PESA (Progression of Early Subclinical Atherosclerosis) study, which objectively measured vigorous recreational physical activity, was associated with higher prevalence of isolated excessive trabeculation. This was double in those making up the highest quintile achieving vigorous physical activity compared to those with no vigorous physical activity.^85^ Such a relationship between increased trabeculation in the setting of cardiac adaptation to exercise, however, is not consistent.

At the levels of physical activity reported by individuals who were nonathletic making up the community-based UK Biobank study, there was no evidence to suggest a dose-response relationship between physical activity intensity and the extent of left ventricular trabeculation.^86^ A prospective study of 68 novice runners, with a mean age of 28, evaluated before and after completion of a marathon, found no change in the extent of ventricular trabeculation.^87^

### Hematological disorders

Excessive trabeculation has been described in up to one-sixth of patients with β-thalassemia, raising the issue of a differential diagnosis between cardiomyopathy with excessive trabeculation and thalassemic cardiomyopathy.^88^, ^89^, ^90^, ^91^ Echocardiographic data of patients with sickle cell disease and Black control subjects who are healthy and asymptomatic revealed a higher prevalence of excessive trabeculation when compared to patients who are normotensive.^92^

Chemotherapy-related cardiac dysfunction is now also emerging as being potentially associated with excessive trabeculation.^93^^,^^94^ The phenotype has been interpreted as a myocardial response to drug toxicity, but it may also be a consequence, rather than the cause, of the cardiac dysfunction.

Ultimately, in patients with hemoglobinopathies and other chronic hematologic disorders, the finding of excessive trabeculation should be interpreted as an adaptive response to increased cardiac preload.^95^ With the majority of individuals exhibiting excessive trabeculation having preserved ventricular function, it is unlikely to represent an underlying myopathic process.

### Renal disorders

A number of reports have described a possible association between polycystic kidney disease and a cardiomyopathy said to be caused by excessive trabeculation.^96^, ^97^, ^98^, ^99^ It remains to be elucidated, however, whether the association can be explained by a genetic interaction between the genes producing polycystic kidney disease and those altered in inherited cardiomyopathies.^96^ An alternative explanation is that excessive trabeculation develops or is unmasked by the increase in cardiac preload known to be associated with chronic renal failure.^100^

## Published Reports of Adverse Consequences of Cardiomyopathy With Excessive Trabeculation

### Arrhythmia

Arrhythmias are common in heart failure, in part because of coexistent myocardial fibrosis.^101^ Most individuals who are symptomatic present with electrocardiographic abnormalities.^102^ Although the complex myocardial architecture of excessive trabeculation might intuitively be linked to a propensity for re-entrant tachycardias, there is no evidence to substantiate this. Indeed, after correction for confounders such as ventricular dilation, systolic dysfunction, and myocardial fibrosis, excessive trabeculation does not appear to confer an additional arrhythmic risk.^103^ The ventricular premature beats noted in patients with excessive trabeculation most often originate from regions of myocardial scar, or from the ventricular outflow tract,^104^ the latter being the least trabeculated portion of the ventricle.^105^

### Thrombus

There are numerous reports on presence of thrombus in the trabecular layer of patients with cardiomyopathy.^10^ However, cohort studies do not support an elevated risk of severe events in the setting of excessive trabeculation,^106^ especially when other parameters are taken into account.^107^^,^^108^ Thrombus lodged between trabeculations, nonetheless, is well documented in symptomatic cases.^10^^,^^109^ Ventricular thrombus is generally associated with cardiomyopathy, myocardial dysfunction, and heart failure,^110^, ^111^, ^112^ which coexist with excessive trabeculation.

### Left ventricular dysfunction

Trabeculations have also been suggested to reduce the compliance of the ventricular wall,^113^^,^^114^ but these hypotheses have not been tested.^37^ In otherwise normal hearts, capillarization and density of sarcomeres and mitochondria are similar in trabeculated and compact myocardium.^115^^,^^116^ Although it is difficult precisely to replicate the anatomy of the trabecular meshwork, modeling of left ventricular function has suggested a positive impact of trabeculation on pump function.^57^ When measured by ejection fraction, studies of human cohorts have revealed either no, or very weak, correlations between the extent of trabecular myocardium and function.^6^^,^^19^^,^^57^ In the UK Biobank, a greater fractal dimension was associated with higher cardiac index.^57^ Some uncertainty remains, however, as in MESA, healthy individuals, but who were in the top quartile for the extent of trabeculation, had slightly reduced circumferential strain when compared to individuals in the lowest quartile.^117^

## Natural History of Cardiomyopathy in the Setting of Excessive Trabeculation

Zemrak et al^19^ evaluated individuals who were asymptomatic in the MESA study with excessive trabeculation over 10 years of follow-up. These investigators assessed excessive trabeculation as a ratio of compact versus trabecular layers, as well as the extent of trabeculation according to the number of segments. Neither factor was associated with adverse cardiac remodeling.^19^ In individuals who are asymptomatic with excessive trabeculation in MESA, there was no relationship between the observed degree of trabeculation and diffuse fibrosis.^117^

Multiple studies have identified the presence of left ventricular dysfunction as the principal mediator for adverse outcomes in the presence of excessive trabeculation.^103^^,^^108^^,^^118^^,^^119^ In these studies, the pooled cardiovascular mortality of individuals with reduced ejection fraction was twice that of those with normal ventricular function. CMR studies using late gadolinium enhancement provide additional prognostic information. A meta-analysis of patients with excessive trabeculation found an increased risk of hard cardiac events in patients with late gadolinium enhancement by CMR.^107^ In the absence of late gadolinium enhancement, or evidence of impaired ventricular function, no hard cardiac events were recorded.^107^ A large single-center study of individuals with excessive trabeculation revealed that survival at 5 years was comparable to an age- and sex-matched population when left ventricular systolic function was preserved.^118^

**Figure 3:**
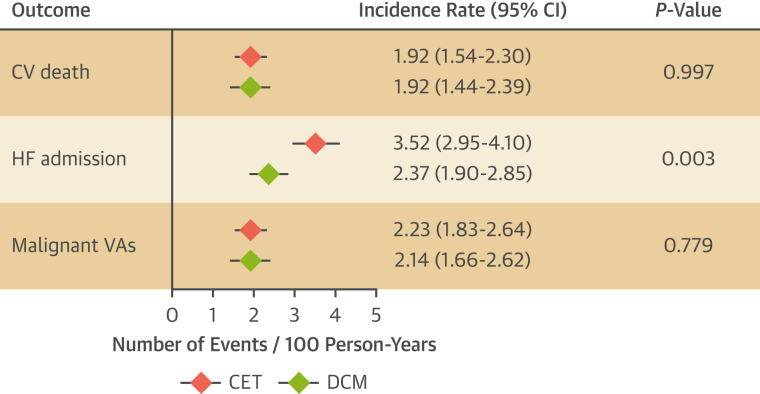
Prognosis of CET and DCM Prognostic comparison between dilated cardiomyopathy (DCM) **(green diamonds)** and cardiomyopathy with excessive trabeculation (CET) **(red diamonds)** based on previously published meta-analyses.^108^^,^^123^ Because conditions of greater preload and afterload are associated with excessive trabeculation, excessive trabeculation as a cause of HF admission should not be inferred from meta-analyses. CV = cardiovascular; HF = heart failure; VA = ventricular arrhythmia.

Few studies have compared the prognosis for patients with cardiomyopathy and excessive trabeculation to that of other nonischemic cardiomyopathies. Among patients with dilated cardiomyopathy, the extent of trabeculation did not influence event-free survival in either unadjusted or adjusted models.^120^ An observational study of patients meeting echocardiographic criteria for excessive trabeculation reported more frequent composite cardiovascular events when compared with age-matched patients with dilated cardiomyopathy,^121^ albeit without considering the role of potential confounders. Another study, after multivariable adjustment over a median follow-up period of 5 years, did not find any difference in event-free survival rate in idiopathic dilated cardiomyopathy vs cardiomyopathy with excessive trabeculation.^122^ Figure 3 is based on previously published meta-analyses,^108^^,^^123^ showing that the pooled event rate of cardiovascular death and malignant ventricular arrhythmias was comparable between dilated cardiomyopathy and cardiomyopathy with excessive trabeculation. A higher incident rate of heart failure hospitalization associated with cardiomyopathy with excessive trabeculation deserves further evaluation in prospective and adequately designed studies.

**Figure 4:**
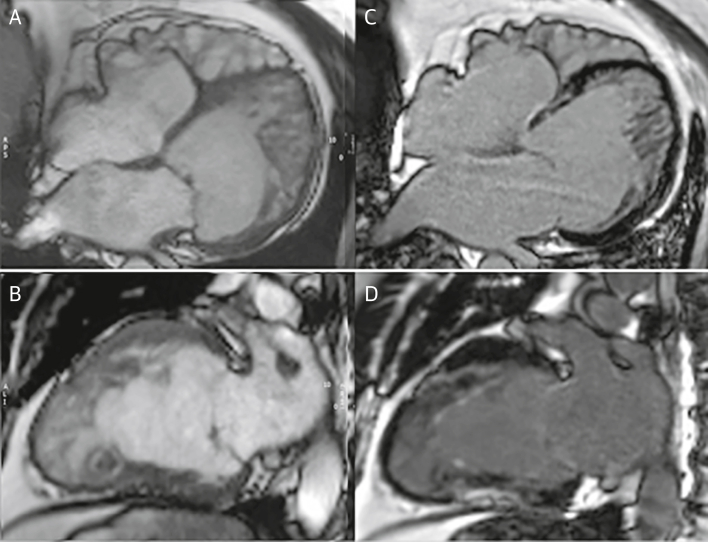
Case Report 1 A 63-year-old man presented with a history of nonsustained ventricular tachycardia and paroxysmal atrial fibrillation treated with atrial ablation. CMR was performed for further evaluation. Cine images at end-diastole in 4-chamber **(A)** (Video 1) and 2-chamber long-axis views **(B)** (Video 2) show excessive trabeculation with biventricular dilation with an EF of 48%. Scattered areas of late gadolinium enhancement were present with a nonischemic pattern **(C and D).** Genetic testing showed a *MYH7* allelic variant. Stress perfusion cardiac magnetic resonance (not shown) showed diffuse perfusion abnormalities in multiple myocardial segments. Although excessive trabeculation is present, the presentation of ventricular dilatation, low EF, and nonischemic myocardial scar and genetic abnormality is the same as in dilated cardiomyopathy. Patient treatment is based on the symptoms and the prognostic risks of arrhythmia, stroke, and contractile impairment. CMR = cardiac magnetic resonance; EF = ejection fraction.

The bulk of current evidence suggests that the phenotypic feature of excessive trabeculation has no independent prognostic relevance in otherwise healthy individuals with no clinical suspicion of inherited cardiac conditions or symptoms. In patients with excessive trabeculation and a known cardiomyopathy, in contrast, the risk for major adverse clinical events appears to be associated with the latter and is apparently independent of the coexisting trabeculation (Figure 4, Videos 1 and 2). Independent prognostic markers include the severity of left ventricular impairment and presence of myocardial injury, rather than the extent of trabeculation.

## Does Excessive Trabeculation Have Different Implications for Children Compared to Adults?

**Figure 5:**
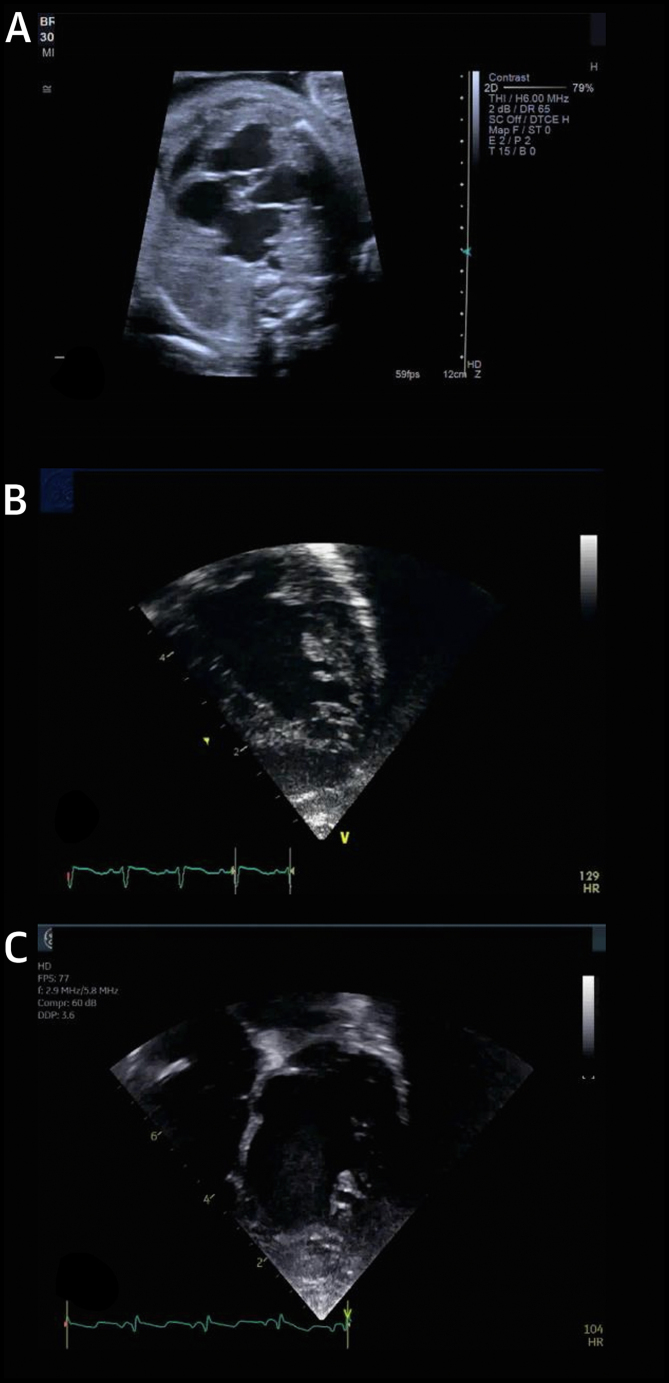
Case Report 2 **(A)** Fetal echocardiogram at 25 weeks in a child with hydrops fetalis demonstrated severely depressed biventricular systolic dysfunction with excessive trabeculation (Video 3)**. (B)** Supraventricular tachycardia in this fetus developed 1 week later. Forty-eight hours after delivery, left ventricular EF reduced to 33% (Video 4)**. (C)** Five years later, the same patient demonstrated progressive left ventricular dilatation with an left ventricular end-diastolic diameter *Z* score of +2.7 and borderline EF of 50% and global longitudinal strain of −17% (Video 5)**.** The working diagnosis was cardiomyopathy with excessive trabeculation. Neonates and children with excessive trabeculation have been understudied, with low rates of longitudinal follow-up. Such patients should routinely undergo follow-up, with close clinical evaluation and potentially neuromuscular disease testing. If familial disease is suspected, genetic testing may be indicated. Abbreviation as in Figure 4.

**Figure 6:**
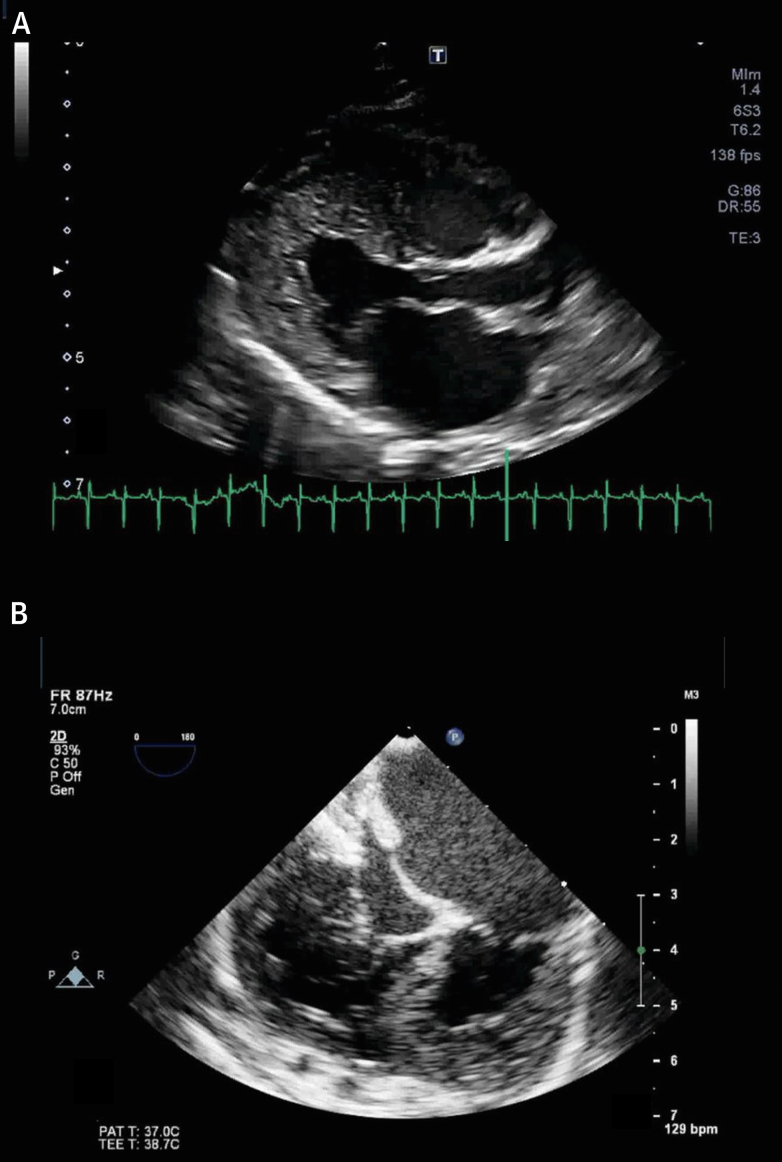
Case Report 3 **(A)** A 3-month-old boy presented with biventricular systolic dysfunction with excessive trabeculation (Video 6)**. (B)** At 6 months of age, this evolved to a restrictive phenotype requiring placement of a left ventricular assist device (Berlin heart) (Video 7)**.** At 12-months of age (not shown), the patient underwent orthotopic heart transplantation. The clinical management and significance of excessive trabeculation and associated disorders in young patients is not well established. As in this example, the evolution of myocardial dysfunction may not be predictable on baseline examination. For patients with myocardial dysfunction in particular, close clinical follow-up is suggested.

Like adults, children with normal ventricular size and function may have excessive trabeculations, frequently representing a normal variant. Congenital heart defects, such as Ebstein malformation and isomerism of the atrial appendages, may coexist with excessive trabeculation and complicate the picture.^124^, ^125^, ^126^, ^127^ Between these extremes of normal variants and overt disease, patients are encountered for whom the extent of trabeculation is neither normal nor markedly abnormal. These patients pose challenges in management, including the uncertain criteria for instigating metabolic and genetic testing, the need for antiplatelet therapy, and the frequency of follow-up (Figures 5 and 6, Video 3, Video 4, Video 5, Video 6, Video 7).

## Does Excessive Trabeculation in Athletes Warrant a Different Approach Than for the Nonathlete?

Using echocardiography, the prevalence of excessive trabeculation fulfilling the criterion for recognition as a potential cardiomyopathy among competitive athletes ranges from around 1%^84^ to just under 10%.^4^ However when using data available from forensic registries, no instances of sudden death in athletes have been directly attributed to excessive trabeculation.^128^, ^129^, ^130^, ^131^, ^132^, ^133^ No adverse cardiac events, furthermore, have been reported in the individuals with normal left ventricular function, regardless of the extent of left ventricular trabeculation.^65^

**Figure 7:**
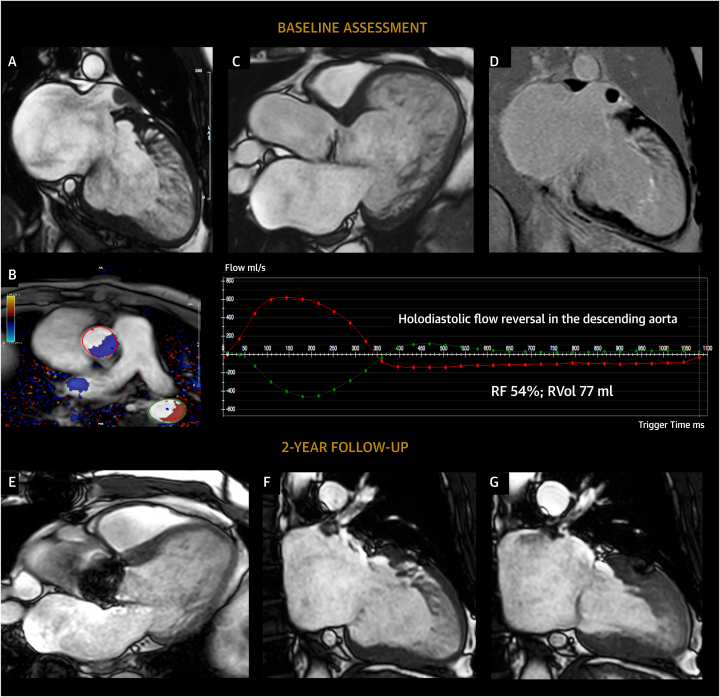
Case Report 4 Images of a 38-year-old master triathlete with history of catheter ablation for persistent atrial fibrillation. Echocardiography at preparticipation screening documented the presence of mildly reduced left ventricular systolic function, mild eccentric aortic regurgitation, and a severely dilated LV with excessive trabeculation (positive Jenni and Chin criteria) that resulted in the diagnosis of so-called left ventricular noncompaction. CMR confirmed the presence of a severely dilated LV with mildly impaired systolic function and excessive trabeculation **(A)** according to the Petersen criteria (noncompacted [trabecular] to compact layer ratio: 3.1). Severe eccentric aortic regurgitation was present **(B and C)** in addition to a thrombus within the left atrial appendage **(A and D).** Six months after surgical aortic valve replacement **(E)** and left atrial appendage occlusion, significant left ventricular reverse remodeling occurred with near-complete normalization of left ventricular function, volumes, and diameters (end-diastolic frame **[F]**; end-systolic frame **[G]**), further leading to a reduction of Petersen criteria for excessive trabeculation (noncompacted [trabecular] to compact layer ratio: 2.4). Moderate-to-severe left ventricular dilatation must be carefully investigated in athletes, irrespective of extent of ventricular trabeculation. If the excessive trabeculation had been part of a cardiomyopathy, arguably, these substantial improvements in left ventricular volumes and function would not have been observed. RF = regurgitant fraction; RVol = regurgitant volume; other abbreviations as in Figures 1 and 4.

Among athletes, excessive trabeculation is of concern in individuals who have either left ventricular systolic dysfunction or dilation, cardiac symptoms, or abnormal electrocardiographic findings unrelated to training (Figure 7). Alternatively, they may have a positive family history of cardiomyopathy.^65^ Since 2013, academic centers for sports cardiology in England ceased to investigate athletes who were asymptomatic with normal cardiac function and normal electrocardiograms, but with echocardiographic criteria for excessive trabeculation.^133^

Athletes with symptoms, abnormal electrocardiographic findings, or a family history of cardiomyopathy should undergo cardiopulmonary exercise testing, Holter monitoring, and CMR.^84^^,^^134^^,^^135^ Balanced atrioventricular remodeling, normal or supranormal indices of diastolic function, normal longitudinal systolic function, and preserved contractility reserve all suggest physiological adaptation to the intensity and/or frequency of physical workload.^135^, ^136^, ^137^ The recommendations for competitive athletes that have features of cardiomyopathy are not altered by the additional presence of excessive trabeculation.^65^^,^^138^ No data exist to indicate that athletes with isolated excessive trabeculation and normal myocardial structure and function should be routinely disqualified from participation in high-intensity exercise and competitive sport.

## Future Research

Controversies remain regarding the clinical significance of excessive trabeculation in both adults and children. For adults, patients who are asymptomatic with excessive trabeculation but normal ventricular function and normal chamber size appear to require little or no long-term follow-up unless other clinical concerns exist, such as family history or electrocardiogram abnormalities. No prospective cohort or registry data are currently available to validate this approach over a 5- or 10-year period. Ongoing collection of outcomes and covariates (including genetic analysis) in large studies such as UK Biobank may prove beneficial to understanding the independent physiologic impact of excessive trabeculation in these otherwise healthy individuals.

For adults with cardiomyopathies combined with excessive trabeculation, questions remain regarding the implications of the trabecular phenotype. As diagnostic and genetic testing has become more sophisticated, the underlying etiology of these and other cases of cardiomyopathy will increasingly be understood. This may result in an ability to make more specific etiologic diagnoses as opposed to a general statement of cardiomyopathy with excessive trabeculation. Large, multi-institutional databases of patients with cardiomyopathy and excessive trabeculation would allow tracking of long-term outcomes, final diagnoses, and potential treatments. Further research to determine whether cardiomyopathy with excessive trabeculation have a different natural history compared to dilated cardiomyopathy without excessive trabeculation may be desirable. Such studies require careful statistical adjustment for confounders, thus increasing complexity.

For neonates, children, and adolescents, the research focus is somewhat different. Longitudinal studies of the early development of the myocardium may shed light on the origin of varying phenotypes. Severe malformations of the trabecular layer, poorly developed left ventricular papillary muscles, along with frequent congenital heart disease and sudden death have been suggested as a distinct cardiomyopathy in neonates and young children.^22^ Although very rare in the older child or adult, improved understanding of any relationship to adult-type excessive trabeculation is important to understand risk of cardiomyopathy and/or sudden death. Heritable links between such patients and those with neuromuscular disorders, other cardiomyopathies, including combinations with excessive trabeculation in the adult, should be explored to help guide management and treatment.

## Conclusions

This review summarizes the evidence and uncertainties regarding the phenotypic feature of excessive trabeculation and its potential associations with cardiomyopathies (Central Illustration). Because the trabeculated myocardium does not coalesce to form the compact myocardial wall, the traditional terminology of left ventricular noncompaction should be discouraged. Excessive trabeculation is frequently detected in the presence of features of a heart muscle disorder, but it is also frequently encountered as a normal variation or develops as a reversible component of physiological cardiac adaptation. The recognition of a highly trabeculated left ventricle is not known to influence prognosis nor management in adults. In neonates and children, caution is warranted because there are multiple reports with genetic abnormalities and neuromuscular disorders in this vulnerable population. Future efforts on characterizing the outcomes and characteristics of those exhibiting excessive trabeculation are warranted.

**Central Illustration:**
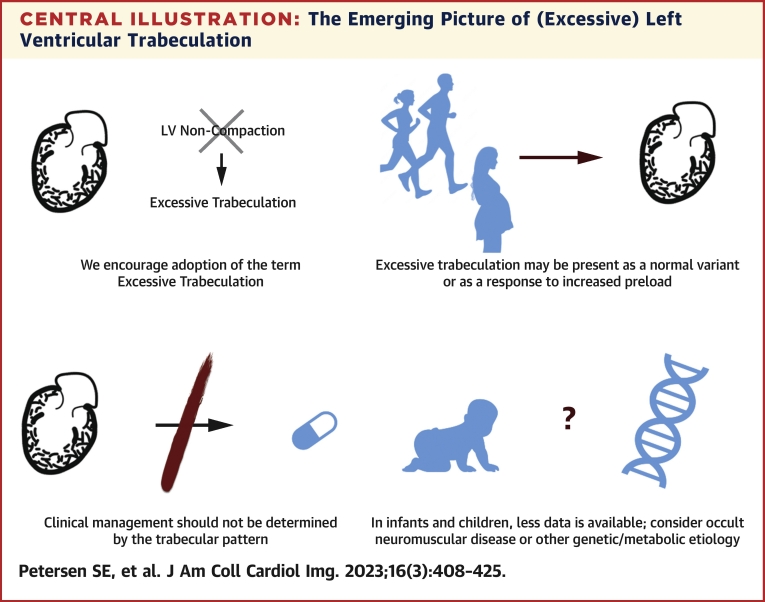
The Emerging Picture of (Excessive) Left Ventricular Trabeculation LV = left ventricular.

## Funding Support and Author Disclosures

Dr Petersen has received support from the National Institute for Health and Care Research Barts Biomedical Research Centre; and has consulted with Circle Cardiovascular Imaging Inc. Dr Friedrich has received support from the McGill Health Centre Foundation; has served as an advisor to and is a shareholder of Circle Cardiovascular Imaging Inc; and is a founder and shareholder of Area19 Medical Inc. All other authors have reported that they have no relationships relevant to the contents of this paper to disclose.
