# Supplementary material for: Excessive Trabeculation of the Left Ventricle: JACC: Cardiovascular Imaging Expert Panel Paper
Source: JACC Cardiovasc Imaging. 2023 Mar;16(3):408–25. doi: 10.1016/j.jcmg.2022.12.026 (PMC9988693; doi:10.1016/j.jcmg.2022.12.026)
Supplement: Supplemental Appendix [file mmc1.docx]

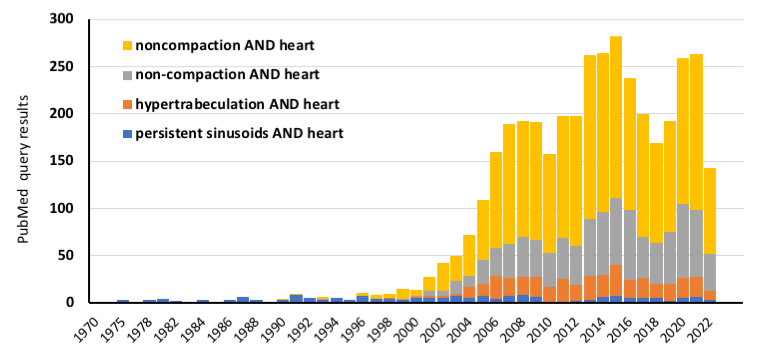


**Supplemental Figure 1:** PubMed query results by year (as of July 7, 2022) on publications related to noncompaction.

**Supplemental Table 1:** So-called noncompaction in the guidelines

| **Guidelines** | **Recommendations and extracts (all quotes from guidelines)** |
| --- | --- |
| 2020 ESC guidelines for the management of adult congenital heart disease^1^ | Altered myocardial architecture (noncompaction) and ventricular interdependence can compromise systolic  ventricular function |
| 2020 ESC guidelines on sports cardiology and exercise in patients with cardiovascular disease^2^ | 5.5.3 Exercise recommendations in individuals with left ventricular noncompaction  LV noncompaction (LVNC) is an unclassified cardiomyopathy characterized by prominent trabeculation and deep recesses that communicate with the LV cavity. Clinical presentation of LVNC includes progressive LV systolic dysfunction, ventricular tachyarrhythmias, and thromboembolic events. Athletes often demonstrate LV hypertrabeculation and up to 8% fulfill the echocardiographic criteria for a diagnosis of LVNC. It is hypothesized that an increased cardiac preload may unmask LV trabecular morphology. Therefore, among athletic individuals, the suspicion of LVNC should only be considered in those who fulfill echocardiographic criteria for LVNC but also have either LV systolic dysfunction (EF <50%), symptoms suggestive of cardiac disease, or a positive family history of LVNC. Additional echocardiographic criteria include a very thin compacted epicardial layer (5 mm in end-diastole on CMR, or <8 mm in systole) and abnormal myocardial relaxation (average E′ < 9 cm/s on tissue Doppler imaging). Such athletes will require further assessment with CMR, exercise echocardiography, and Holter monitor to assess the presence of LV fibrosis, cardiac thrombi, contractile reserve, and exercise-induced complex arrhythmias.  5.5.3 Exercise recommendations in individuals with left ventricular noncompaction  5.5.3.1 Risk stratification  The clinical outcomes of LVNC are determined by the presence of symptoms, severity of LV dysfunction, and the nature of the VAs. There are no reported adverse cardiac events in the absence of LV dysfunction regardless of the severity of LV trabeculation.  5.5.3.2 Follow-up  Regular follow-up is recommended for individuals with LVNC. New symptoms should prompt interruption of exercise and re-evaluation.  Recommendations for exercise in individuals with left ventricular noncompaction cardiomyopathy  Recommendation for diagnosis:  A diagnosis of LVNC in athletic individuals should be considered if they fulfill imaging criteria, in association with cardiac symptoms, family history of LVNC or cardiomyopathy, LV systolic (EF <50%) or diastolic (E <9 cm/s) dysfunction, a thin compacted epicardial layer (<5 mm in end-diastole on CMR, or <8 mm in systole on echocardiography), or abnormal 12-lead ECG. [Class IIa, Level of Evidence: B]  Exercise recommendations:  Participation in high-intensity exercise and all competitive sports, if desired, with the exception where syncope may cause serious harm or death, may be considered in asymptomatic individuals with LVNC and LVEF ≥50% and absence of frequent and/or complex VAs. [Class IIb, Level of Evidence: C]  Participation in recreational exercise programs of low to moderate intensity, if desired, may be considered in individuals with LVEF of 40%-49% in the absence of syncope and frequent or complex VAs on ambulatory Holter monitoring or exercise testing. [Class IIb, Level of Evidence: C]  Participation in high- or very high-intensity exercise including competitive sports, if desired, may be considered for individuals who are gene positive for LVNC but phenotype negative (with the exception of lamin A/C or filamin C carriers). [Class IIb, Level of Evidence: C]  Participation in high-intensity exercise or competitive sports is not recommended in individuals with any of the following: symptoms, LVEF <40% and/or frequent and/or complex VAs on ambulatory Holter monitoring or exercise testing. [Class III, Level of Evidence: C]  Follow-up and further considerations:  Annual assessment for risk stratification is recommended for individuals with LVNC and genotype-positive/phenotype-negative individuals who exercise on a regular basis. [Class I, Level of Evidence: C] |
| 2021 ESC guidelines for the diagnosis and treatment of acute and chronic heart failure^3^ | Recommendations for specialized diagnostic tests for selected patients with chronic heart failure to detect reversible/treatable causes of heart failure:  CMR:  CMR is recommended for the characterization of myocardial tissue in suspected infiltrative disease, Fabry disease, inflammatory disease (myocarditis), LV noncompaction, amyloid, sarcoidosis, iron overload/hemochromatosis. [Class I, Level of Evidence: C]  In addition, CMR allows myocardial characterization in, eg, myocarditis, amyloidosis, sarcoidosis, Chagas disease, Fabry disease, LV noncompaction CMP, hemochromatosis, and arrhythmogenic cardiomyopathy (AC).  Patients with visible intraventricular thrombus or at high thrombotic risk, such as those with history of peripheral embolism or some patients with PPCM or LV noncompaction (LVNC), should be considered for anticoagulation.  14.3 Left ventricular noncompaction  LVNC is a very rare congenital CMP characterized by endomyocardial trabeculations that increase in number and prominence. In most cases, including when the condition is caused by mutations in the MYH7 or MYBPC3 gene, LVNC is inherited in an autosomal dominant pattern.  A clear overlap exists in families with DCM and HCM phenotypes. Quite commonly individuals with features of LVNC are found in families where other affected relatives have typical HCM or DCM. Therefore, LVNC is not treated as a separate disease entity, but as a separate rare presentation of a genetic susceptibility to either HCM or DCM.  The pathophysiology of cardiac dysfunction in ACHD is often different from noncongenital (acquired) heart disease, in particular in those with: a systemic right ventricle (RV), a failing subpulmonary ventricle, a single ventricle, surgery-related injury, chronic pressure/volume overload in systemic and subpulmonary ventricles, and those with hypertrophy or noncompaction induced by gene mutation. |
| 2022 ESC guidelines on cardiovascular assessment and management of patients undergoing noncardiac surgery: Developed by the task force for cardiovascular assessment and management of patients undergoing non-cardiac surgery of the European Society of Cardiology (ESC) Endorsed by the European Society of Anaesthesiology and Intensive Care (ESAIC)^4^ | 3.3.1.1. Patients aged <65 years without a history of cardiovascular disease or cardiovascular risk factors  Patients without signs or symptoms of CVD, but with a family history of genetic cardiomyopathy (ie, dilatated, hypertrophic, arrhythmic, or restrictive cardiomyopathy, or LV noncompaction) should be evaluated with an ECG and an echocardiographic examination to rule out the presence of the disease, irrespective of the age. |
| 2022 ESC guidelines for the management of patients with ventricular arrhythmias and the prevention of sudden cardiac death: Developed by the task force for the management of patients with ventricular arrhythmias and the prevention of sudden cardiac death of the European Society of Cardiology (ESC). Endorsed by the Association for European Paediatric and Congenital Cardiology (AEPC)^5^ | 7.1.3.4. Left ventricular noncompaction  LVNC comprises a heterogeneous group of diseases. The diagnosis is challenging, and various diagnostic criteria have been proposed. The yield of genetic tests in index patients is low.  The morphological phenotype of noncompaction based on imaging parameters may also appear in a healthy population.  A meta-analysis including 2,501 LVNC patients revealed a risk of cardiovascular mortality similar to that of DCM patients, with no relation to the extent of trabeculation. CMR-based detection of focal fibrosis using LGE in LVNC with preserved ejection was associated with serious cardiac events (aborted death, ICD therapy, heart transplantation [HTX]/LVAD) in another meta-analysis including 574 patients (OR: 6.1; 95% CI: 2.1-17.5; *P* < 0.001). A combination of CMR criteria with systematic genotyping may overcome current uncertainties regarding risk stratification.  Recommendation Table 31—Recommendations for implantable cardioverter-defibrillator implantation in left ventricular noncompaction  In patients with a LVNC cardiomyopathy phenotype based on CMR or echocardiography, implantation of an ICD for primary prevention of SCD should be considered to follow DCM/HNDCM recommendations. [Class IIa, Level of Evidence: C]  5.1.3.4. Imaging  Imaging is crucial to assess cardiac function and detect cardiomyopathies. A negative imaging study supports primary electrical disease in a patient with VA. Echocardiography is a readily available and first-line diagnostic and risk stratification tool for valve diseases, CAD and DCM, HCM, ARVC, and left ventricular noncompaction (LVNC).  CMR currently provides the most accurate and reproducible measurement of atrial, biventricular global and regional systolic function, and can detect myocardial edema, fibrosis, infiltration, and perfusion defects. CMR is more sensitive than echocardiography to diagnose ARVC, is diagnostic in LVNC, and can detect apical aneurysms in HCM. |
| ACC/AHA/HRS 2008 guidelines for device-based therapy of cardiac rhythm abnormalities^6^ | 3.2. Primary Prevention of Sudden Cardiac Death  Primary prevention of SCD refers to the use of ICDs in individuals who are at risk for but have not yet had an episode of sustained VT, VF, or resuscitated cardiac arrest. Clinical trials have evaluated the risks and beneﬁts of the ICD in prevention of sudden death and have improved survival in multiple patient populations, including those with prior MI and heart failure caused by either coronary artery disease or nonischemic DCM. Prospective registry data are less robust but still useful for risk stratiﬁcation and recommendations for ICD implantation in selected other patient populations, such as those with HCM, ARVD/C, and the long-QT syndrome. In less common conditions (eg, Brugada syndrome, catecholaminergic polymorphic VT, cardiac sarcoidosis, and LV noncompaction), clinical reports and retrospectively analyzed series provide less rigorous evidence in support of current recommendations for ICD use, but this constitutes the best available evidence for these conditions.  3.2.6. Noncompaction of the Left Ventricle  Noncompaction of the LV is a rare congenital cardiomyopathy characterized anatomically by excessive prominent trabeculae and deep intertrabecular recesses in the LV without other major congenital cardiac malfunction. The origin of the anatomic abnormalities is likely caused by an arrest of normal embryogenesis of the endocardium and epicardium of the ventricle during development. This leads to suspension of the normal compaction process of the loose myocardial meshwork. Diagnosis is difﬁcult and is frequently missed or delayed because of lack of knowledge about this uncommon disease. Echocardiography is considered by many to be the diagnostic procedure of choice, but some cases are detected by computed tomography or cardiac magnetic resonance. Abnormalities in the resting ECG, including bundle-branch block or ST-segment depression, are found in most patients, but the ﬁndings do not have a high degree of sensitivity or speciﬁcity.  Ventricular arrhythmias and sudden death are among the major complications of this disorder. Sudden death can occur at any age, and there are currently no techniques clinically useful for risk stratiﬁcation for life-threatening ventricular arrhythmias with noncompaction. Although there is no impairment of systolic function, ventricular arrhythmias are frequent in noncompaction. Approximately 40% of children with noncompaction demonstrate complex ventricular arrhythmias. Available clinical data indicate that sudden death is the most common cause of mortality. Although there are no prospective trials or registry data, there are sufﬁcient observational data to indicate that placement of an ICD as a strategy to reduce the risk of sudden death is a reasonable clinical strategy. |
| 2017 ACC/AHA/HRS guideline for the evaluation and management of patients with syncope^7^ | Conditions uncommonly associated with syncope:  LV noncompaction. Clinical Characteristics: Cardiomyopathy characterized by prominent LV trabeculae and deep intertrabecular recesses, caused by embryological perturbation. Notes: Syncope reported in 5%-9% of both adult and pediatric patients. The mechanism may be tachyarrhythmia.  Recommendations for athletes:  Assessment by a specialist with disease-specific expertise is reasonable for athletes with syncope and high-risk markers. [Class IIa, Level of Evidence: C]  Syncope in the competitive athlete requires an evaluation for potentially fatal causes of syncope, especially when evidence of HCM, LQTS, Wolff-Parkinson-White syndrome, ARVC, ventricular noncompaction, symptomatic mitral valve prolapse, Marfan syndrome, congenital coronary anomalies, or other at-risk conditions is present. Any suspected cardiovascular pathology requires further evaluation, and family counseling and/or genetic testing is advised for those conditions with a known familial tendency. |
| 2022 AHA/ACC/HFSA guideline for the management of heart failure^8^ | CMR provides noninvasive characterization of the myocardium that may provide insights into HF cause. Late-gadolinium enhancement, reflecting fibrosis and damaged myocardium, can identify acute and chronic MI and identify HF caused by CAD. Patterns of late-gadolinium enhancement or specific T-1 and T-2 techniques can suggest specific infiltrative and inflammatory cardiomyopathies, such as myocarditis, sarcoidosis, Fabry disease, Chagas disease, noncompaction, iron overload, and amyloidosis. |

Abbreviations used in the guidelines listed: AC, arrhythmogenic cardiomyopathy; ACC, American College of Cardiology; ACHD, adult congenital heart disease; AHA, American Heart Association; ARVC, arrhythmogenic right ventricular cardiomyopathy; CAD, coronary artery disease; CMP, cardiomyopathy; CMR, cardiac magnetic resonance; CVD, cardiovascular disease; DCM, dilated cardiomyopathy; ECG, electrocardiogram; EF, ejection fraction; ESC, European Society of Cardiology; HCM, hypertrophic cardiomyopathy; HF, heart failure; HNDCM, hypokinetic nondilated cardiomyopathy; HTx/LVAD, heart transplant/left ventricular assist device; ICD, implantable cardioverter-defibrillator; LGE, late gadolinium enhancement; LQTS, long QT syndrome; LV, left ventricle; LVNC, left ventricular noncompaction; MI, myocardial infarction; PPCM, peripartum cardiomyopathy; SCD, sudden cardiac death; VA, ventricular arrhythmias; VF, ventricular fibrillation; VT, ventricular tachycardia.

**Supplemental Table 1 References**

1. Baumgartner H, De Backer J, Babu-Narayan SV, et al. 2020 ESC guidelines for the management of adult congenital heart disease. *Eur Heart J*. 2021;42:563-645.

2. Pelliccia A, Sharma S, Gati S, et al. 2020 ESC guidelines on sports cardiology and exercise in patients with cardiovascular disease. *Eur Heart J.* 2021;42:17-96.

3. McDonagh TA, Metra M, Adamo M, et al. 2021 ESC guidelines for the diagnosis and treatment of acute and chronic heart failure. *Eur Heart J.* 2021;42:3599-3726.

4. Halvorsen S, Mehilli J, Cassese S, et al. 2022 ESC guidelines on cardiovascular assessment and management of patients undergoing non-cardiac surgery. *Eur Heart J.* 2022;43:3826-3924.

5. Zeppenfeld K, Tfelt-Hansen J, de Riva M, et al. 2022 ESC guidelines for the management of patients with ventricular arrhythmias and the prevention of sudden cardiac death. *Eur Heart J.* 2022;43:3997-4126.

6. Epstein AE, DiMarco JP, Ellenbogen KA, et al. ACC/AHA/HRS 2008 guidelines for device-based therapy of cardiac rhythm abnormalities: a report of the American College of Cardiology/American Heart Association Task Force on Practice Guidelines (Writing Committee to Revise the ACC/AHA/NASPE 2002 Guideline Update for Implantation of Cardiac Pacemakers and Antiarrhythmia Devices) developed in collaboration with the American Association for Thoracic Surgery and Society of Thoracic Surgeons. *J Am Coll Cardiol.* 2008;51:e1-e62.

7. Shen WK, Sheldon RS, Benditt DG, et al. 2017 ACC/AHA/HRS guideline for the evaluation and management of patients with syncope: a report of the American College of Cardiology/American Heart Association Task Force on Clinical Practice Guidelines and the Heart Rhythm Society. *J Am Coll Cardiol*. 2017;70:e39-e110.

8. Heidenreich PA, Bozkurt B, Aguilar D, et al. 2022 AHA/ACC/HFSA guideline for the management of heart failure: executive summary: a report of the American College of Cardiology/American Heart Association Joint Committee on Clinical Practice Guidelines. *J Am Coll Cardiol*. 2022;79:1757-1780.

**Experimental models of excessive trabeculation**

There is no naturally occurring model of excessive trabeculation with cardiomyopathic features. Many animals naturally have more left ventricular trabeculation (proportionally) than humans, include other primates (Shave et al 2019). These animals have normal pump function and, at best, can be considered models of the asymptomatic, or “benign,” setting of excessive trabeculation (Finsterer et al 2017). Cardiac ventricles of cold-blooded vertebrates such as fish, amphibians, and reptiles are extremely trabeculated and much more so than even the most extreme cases of human excessive trabeculation (Jensen et al 2016). Model organisms then are mostly those where an experimental perturbation to normal ventricular development can be induced, either with small molecule interventions or genetic manipulation. In effect, model organisms of excessive trabeculation, which was recently reviewed (Purevjav et al 2021), mostly are zebrafish and in particular mouse. Note that the zebrafish ventricle is intrinsically much more trabeculated than the human ventricle and has an extremely thin compact wall (approximately 100 µm), but because of evolutionary conservation it lends itself well to the study of effects of gene knock-outs and introduced mutations (Bakkers 2011). A comprehensive review of the genes associated with normal development of the ventricular wall was published by Wilsbacher and McNally (2016). The extent to which the embryonic ventricular walls form trabeculations is much under the influence of endocardial signaling and this topic is comprehensively reviewed in MacGrogan et al (2018). Some mouse models exhibit extremely trabeculated ventricles, ie, they are more trabeculated than what occurs in any normal state (Gruber et al 1996; Rhee et al 2018; Sandireddy et al 2019). In most of these models, however, the mice die before birth. Humanized mouse, where a patient mutation is inserted, has also been made (eg, Luxán et al 2013), but this model also has a ventricular septal defect (Captur et al 2016) and excessive trabeculation with cardiomyopathy only is rare. Because the embryonic ventricle is naturally more trabeculated (proportionally) than the fetal heart, mouse models with embryonic lethality (die-off before embryonic day 14.5) can hardly be called models of excessive trabeculation.

**Supplemental References**

Bakkers J. Zebrafish as a model to study cardiac development and human cardiac disease. *Cardiovasc Res.* 2011;91(2):279-288. PMID: 21602174

Captur G, Wilson R, Bennett MF, et al. Morphogenesis of myocardial trabeculae in the mouse embryo*. J Anat*. 2016;229(2):314-325. PMID: 27020702

Finsterer J, Stollberger C, Towbin JA. Left ventricular noncompaction cardiomyopathy: cardiac, neuromuscular, and genetic factors. *Nat Rev Cardiol.* 2017;14:224-237.

Gruber PJ, Kubalak SW, Pexieder T, Sucov HM, Evans RM, Chien KR. RXR alpha deficiency confers genetic susceptibility for aortic sac, conotruncal, atrioventricular cushion, and ventricular muscle defects in mice. *J Clin Invest*. 1996;98(6):1332-1343. PMID: 8823298

Jensen B, Agger P, de Boer BA, et al. The hypertrabeculated (noncompacted) left ventricle is different from the ventricle of embryos and ectothermic vertebrates. *Biochim Biophys Acta*. 2016;1863:1696-1706.

Luxán G, Casanova JC, Martínez-Poveda B, et al. Mutations in the NOTCH pathway regulator MIB1 cause left ventricular noncompaction cardiomyopathy. *Nat Med*. 2013;19(2):193-201. PMID: 23314057

MacGrogan D, Münch J, de la Pompa JL. Notch and interacting signalling pathways in cardiac development, disease, and regeneration. *Nat Rev Cardiol*. 2018;15(11):685-704. PMID: 30287945

Purevjav E, Chintanaphol M, Orgil BO, Alberson NR, Towbin JA. Left ventricular noncompaction cardiomyopathy: from clinical features to animal modeling. In: *Preclinical Animal Modeling in Medicine*. IntechOpen; 2021.

Rhee S, Chung JI, King DA, et al. Endothelial deletion of Ino80 disrupts coronary angiogenesis and causes congenital heart disease. *Nat Commun*. 2018;9(1):1-16. PMID: 29371594

Sandireddy R, Cibi DM, Gupta P, et al. Semaphorin 3E/PlexinD1 signaling is required for cardiac ventricular compaction. *JCI Insight*. 2019;4(16):e125908. PMID: 31434798

Shave RE, Lieberman DE, Drane AL, et al. Selection of endurance capabilities and the trade-off between pressure and volume in the evolution of the human heart. *Proc Natl Acad Sci U S A.* 2019;116(40):19905-19910. PMID: 31527253

Wilsbacher L, McNally EM. Genetics of cardiac developmental disorders: cardiomyocyte proliferation and growth and relevance to heart failure. *Annu Rev Pathol*. 2016;11:395-149. PMID: 26925501
